# Male breast cancer: a rare case of neoplasia in elderly; our experience and review of the literature

**DOI:** 10.1186/1471-2482-13-S1-A39

**Published:** 2013-09-16

**Authors:** Stefano Reggio, Luciano Grimaldi, Mario Pannullo, Marco Ferretti, Rita Compagna, Bruno Amato, Michele Danzi

**Affiliations:** 1 Department of Specialized Surgery, Division of Gastrointestinal Surgery Rehabilitation of Election and Emergency. “Federico II” University, Naples, Italy

## Background

Male breast cancer (MBC) is a rare tumor and accounts for <1 % of all breast cancers, but its incidence is increasing. The majority of breast lesions in men are benign and the causes, optimal treatments, and medical/psychosocial sequelae of breast cancer in men are poorly understood. [1] The natural history and prognostic factors do not differ from the female form, this tumor is characterized by a higher mortality rate because the presenting symptoms are often underestimated, and it comes to diagnosis when the disease is already advanced [2].

## Methods

In our study we retrospectively analyzed the clinicopathologic features and prognosis of 16 cases of male breast cancer diagnosed in our hospital from 1998 to 2010, and we assessed whether prognostic factors in male (MBC) and female (FBC) breast cancer have similar impact on survival: the aim of our study was to compare overall survival (OS) and disease-free survival (DFS) in a group of matched males and females with breast cancer with similar prognostic factors (including age, nodal status, resection margin, use of hormonal therapy, chemotherapy with/without hormone and radiation therapy).

## Results

Matching was conducted based on age, year of diagnosis, stage of the disease and pathology. The mean age at diagnosis was 60.0±11.3 years for males and 56.1±10.6 years for females, and the median follow-up time was 54 months (range, 5-242 months) for males and 60 months (range, 29-263 months) for females. The invasive ductal carcinoma in MBC was much higher than in FBC. The positive rate of estrogen receptors in MBC patients was higher than in FBC patients (P = 0.048), whereas HER-2 was positive in 13.8 % of MBC patients, which was significantly lower than in FBC patients (P = 0.001); HER-2-positive patients had a statistically worse prognosis than HER-2-negative patients (P = 0.040). The overall survival rates of MBC were significantly higher than FBC. Lymph node metastases and larger tumor size were also associated with poorer prognosis. Not statistically significant differences were identified for tumor location between the two groups.

## Conclusions

BRCA2 mutations, lower age, lymph nodes metastases and tumor size are proven risk factors. The FBC patients were significantly different from the MBC in clinicopathologic and prognostic characteristics: the HER-2 positivity, much higher in women, was an important factor for bad prognosis [3,4] . Disease biology is distinct in men, but diagnostic approaches and treatments for men are generally extrapolated from those in women due to inadequate research in men.
